# A retrospective analysis of hearing after cholesteatoma surgery: the bony obliteration tympanoplasty versus canal wall up and canal wall down without mastoid obliteration

**DOI:** 10.1007/s00405-022-07367-x

**Published:** 2022-04-10

**Authors:** Hylke F. E. van der Toom, Marc P. van der Schroeff, Mick Metselaar, Anne van Linge, Jantien L. Vroegop, Robert J. Pauw

**Affiliations:** Department of Otorhinolaryngology and Head and Neck Surgery, Erasmus Medical Center, Doctor Molewaterplein 40, 3015 GD Rotterdam, The Netherlands

**Keywords:** Cholesteatoma, Bony obliteration tympanoplasty, Canal wall up, Canal wall downhearing, Udiological results BOT, CWU, CWD

## Abstract

**Objective:**

To evaluate and compare the hearing outcome after the bony obliteration tympanoplasty (BOT), canal wall up (CWU) without mastoid obliteration and canal wall down (CWD) without mastoid obliteration in a large patient cohort. As the aeration of the middle ear is associated with hearing outcome, we hypothesized that the post-operative hearing after the BOT may be better compared to CWU and CWD without obliteration.

**Methods:**

This is a retrospective cohort study on all adult patients who underwent the BOT, CWU without obliteration or CWD without obliteration for primary or revision cholesteatoma between January 2003 and March 2019 with audiological follow-up at our institution. Pre-operative, short-term post-operative and long-term post-operative hearing tests were analyzed and potential factors influencing post-operative hearing were assessed.

**Results:**

626 ears were included. We found no significant differences between the short-term and long-term post-operative audiometry. The pre-operative air–bone gap (ABG) was the factor with the largest effect size on change in air–bone gap (ABG) between pre- and post-operative. When stratifying for this factor along with the type of ossicular chain reconstruction to account for differences at baseline, no significant differences in post-operative ABG were found between BOT and non-obliteration CWU and CWD.

**Conclusion:**

In this large retrospective cohort study, we found no significant differences in post-operative ABG between the BOT and the non-obliteration CWU and CWD. A solid comparison of hearing between groups remains very challenging as hearing outcome seems to be dependent on many different factors. Hearing outcome seems to be no additional argument to choose for BOT over non-obliteration surgery.

**Supplementary Information:**

The online version contains supplementary material available at 10.1007/s00405-022-07367-x.

## Introduction

Although the primary goal of cholesteatoma surgery is complete disease removal with the lowest possible recurrent cholesteatoma rates, optimal post-operative hearing is an important secondary goal and paramount to the patient involved [1]. It is hard to offer reliable individual patient counseling on post-operative hearing after cholesteatoma surgery as post-operative hearing is not only dependent on factors such as the ossicular chain status, ossicular chain reconstruction [2–4] and extension of cholesteatoma [5], but it is also suggested that factors as the aeration of the middle ear [6–8] are associated with the hearing outcome.

Obliteration of the mastoid and epitympanic space is becoming a more and more established technique in cholesteatoma surgery with promising outcome concerning recurrent and residual cholesteatoma rates [9]. It is stated that mastoid obliteration causes a reduction in mucosal surface for gas exchange and that it, therefore, can improve the gas pressure balance in the middle ear resulting in slower gas absorption and slower pressure changes [10] and that mastoid obliteration facilitates better aeration of the middle ear [11]. Since post-operative hearing after cholesteatoma surgery is influenced, among other things, by aeration of the middle ear, we hypothesize that the hearing results after mastoid obliteration in cholesteatoma surgery may be better compared to non-obliterative techniques. While many previous researchers have reported on post-operative hearing after cholesteatoma surgery, there is no study that compares mastoid obliteration to non-obliterative techniques with respect to post-operative hearing.

In our institution, the canal wall up with mastoid obliteration (bony obliteration tympanoplasty, BOT) was introduced in 2013 and gradually replaced the canal wall up (CWU) and canal wall down (CWD) approach without mastoid obliteration. As these different surgical techniques were performed by the same surgeons in the same institution, it is interesting to compare the audiological results of these approaches. Therefore, the aim of this study was to retrospectively compare the audiological results after BOT to those after CWU and CWD without mastoid obliteration in our cohort and to compare our results with literature.

## Materials and methods

### Patients

A retrospective cohort study was conducted at the department of Otorhinolaryngology and Head and Neck Surgery of the Erasmus Medical Center (tertiary referral center) with approval of the medical research ethical committee. All adult patients (≥ 18 years) who underwent the BOT, CWU without mastoid obliteration or CWD without mastoid obliteration for primary or recurrent cholesteatoma between January 2003 and March 2019 were included. Cases without audiological follow-up or with other surgical techniques than mentioned above (i.e., partial mastoid obliteration) were excluded. As the BOT was introduced in 2013 in our institution, all included cases before this introduction consist of CWU and CWD without obliteration. As the BOT was gradually introduced, the included cases from the year 2013 onwards consist of both BOT and the non-obliteration approaches.

### Methods

Patient demographics, surgical technique, primary versus revision surgery, audiological results (as described below), extension of cholesteatoma according to the STAMCO classification [12], ossicular chain status before and after cholesteatoma removal, type of middle ear reconstruction and most recent follow-up date were collected from electronic patient records. The surgical technique was classified as either (1) BOT (2) CWU without obliteration or (3) CWD without obliteration. The ossicular chain status before and after surgery was classified as (1) intact chain, (2) incus absent with intact stapes, (3) stapes superstructure absent with or without intact incus, or (4) footplate fixation. The type of middle ear reconstruction was classified as (1) no reconstruction due to intact chain, (2) no reconstruction due to unfavorable middle ear aspects, (3) incus interposition, (4) partial ossicular replacement prosthesis (PORP), (5) tympanic membrane to stapes (type III tympanoplasty), (6) total ossicular replacement prosthesis (TORP), or (7) tympanic membrane directly to footplate (type IV tympanoplasty).

### Surgical technique

The BOT, CWU and CWD were performed under general anesthesia. The BOT was performed as described by Offeciers [13] and van Dinther et al. [14, 15]. Two grams of cefazolin was administrated intravenously prior to surgery and if necessary repeated after 4 h. After a wide retro-auricular incision, cortical bone chips and cortical bone dust were harvested and stored in a rifamycin solution (Sanofi-Aventis U.S. LLC, Bridgewater, New Jersey, USA. Rifadin® 600 mg powder for infusion with 10 mL solvent for solution and 20 mL 0.9% sodium chloride). A cortical mastoidectomy was performed leaving the posterior canal wall intact, followed by a wide posterior tympanotomy and epitympanotomy. If still present, the malleus head and incus were removed. After removal of all cholesteatoma and accessible cell tracts, bone chips were used to separate the epitympanum and mastoid from the middle ear. The cavity was filled with autologous bone pate and/or bioactive glass granules S53P4 (Bonealive®, Bonalive Biomaterials Ltd., Turku, Finland). If possible an ossicular chain reconstruction was performed using either autologous material (incus interposition or cartilage graft) or a titanium prosthesis. The tympanic membrane was reconstructed using cartilage or temporal fascia. Finally, the ear canal was packed with a gauze with hydrocortisone/oxytetracycline/polymixin B for at least 1 week. In CWU and CWD without mastoid obliteration, usually no antibiotics were administrated prior to surgery. A cortical mastoidectomy was performed with preservation of the posterior canal wall for CWU surgery and removal of the posterior canal wall in cases of CWD surgery. When necessary in CWU surgery, the scutum was reconstructed with bone chips or cartilage. If possible an ossicular chain reconstruction was performed and the tympanic membrane was reconstructed with cartilage or temporal fascia. As with the BOT, the ear canal or mastoid bowl was packed with a gauze with hydrocortisone/oxytetracycline/polymixin B for at least 1 week.

### Audiological evaluation

For each case, the pure tone averages (PTA) calculated over 0.5, 1, 2 and 4 kHz for the air conduction (AC) and bone conduction (BC) threshold were obtained from the electronic patient records from the pre-operative and short-term post-operative hearing test (≥ 6 weeks post-surgery) and if available from the long-term post-operative hearing test (≥ 12 months post-surgery). All thresholds were measured according to the shortened ascending method based on ISO standard 8253–1, which means that thresholds were defined by the intensity level at which the tone was heard in 2 out of 3 ascents. All testing was performed in a sound-attenuated booth at our outpatient department, on clinical audiometers (Decos audiology workstation), calibrated according to international standards. Threshold levels beyond the maximum stimulus level of the audiometer were registered as 120 dB HL.

### Statistical analyses

Statistical analyses were performed using IBM SPSS Statistics 25 (SPSS Inc., Chicago, IL, USA) and GraphPad Prism 8.4.3 (GraphPad Software, San Diego, California, USA). Because of not normally distributed data, the Kruskal–Wallis H test was used to assess for significant differences between the three surgical techniques. The Wilcoxon signed rank test was used to calculate the effect size of different variables on change in ABG based on the Z-score of this test. An effect size between 0.10 and 0.30 was indicated as a small effect size, 0.30–0.50 as a medium effect size, and > 0.50 as a large effect size [16]. The Mann–Whitney U test was used to assess for differences in post-operative ABG between the BOT and non-obliteration techniques. P-values less than 0.05 were considered statistically significant.

### Subgroup analyses

To address the heterogenicity between different groups, variables with a relatively large effect size or clinical relevance on change in ABG were determined. We stratified for these variables in a subgroup analyses for differences in audiological outcome between the BOT and non-obliteration techniques. As the ossicular chain is per definition not intact after the BOT (i.e., the malleus head and incus are removed), cases in the CWU and CWD group with an intact chain after cholesteatoma removal were excluded for this subgroup analyses, facilitating the fairest possible comparison between the surgical techniques.

## Results

Between January 2003 and March 2019, 626 ears of 558 patients underwent a BOT, CWU without obliteration or CWD without obliteration for cholesteatoma with audiological follow-up at our institution. Short-term audiometry was available for all 626 patients and long-term audiometry was available for 560 patients. The median time from surgery to hearing test was 14 weeks (IQR 11–20 weeks) for the short-term audiometry and 20 months (IQR 12–51 months) for the long-term audiometry. One-hundred and ninety-eight (31.6%) cases consisted of BOT, 275 (43.9%) consisted of CWU without obliteration and 153 (24.5%) consisted of CWD without obliteration. In the BOT group 73 cases (36.9%) consisted of primary surgery and 125 (63.1%) revision surgery; in the CWU without obliteration group these numbers were 148 (53.8%) and 127 (46.2%), respectively, and in the CWD without obliteration group, these numbers were 97 (63.4%) and 56 (36.6%), respectively. Patient characteristics per surgical technique are shown in Table 1.

**Table 1:** Patient characteristics per surgical technique

		BOT (*N*, %)	CWU without obliteration (*N*, %)	CWD without obliteration (*N*, %)
All cases		198 (31.6%)	275 (43.9%)	153 (24.5%)
Sex	Female	69 (34.8%)	93 (33.8%)	69 (45.1%)
	Male	129 (65.2%)	182 (66.2%)	84 (54.9%)
Side	Left	100 (50.5%)	136 (49.8%)	82 (53.6%)
	Right	98 (49.5%)	139 (50.5%)	71 (46.4)
Primary/revision	Primary	73 (36.9%)	148 (53.8%)	97 (63.4%)
	Revision	125 (63.1%)	127 (46.2%)	56 (36.6)
Ossicular chain before cholesteatoma removal	Intact chain	18 (9.1%)	53 (19.3%)	13 (8.5%)
	Incus absent	114 (57.6%)	172 (62.5%)	87 (56.9%
	Stapes absent	55 (27.8%)	45 (16.4%)	42 (27.5%)
	Footplate fixation	2 (1.0%)	2 (0.7%)	2 (1.3%)
	Missing	9 (4.5%)	3 (1.1%)	9 (5.9%)
Ossicular chain after cholesteatoma removal	Intact chain	0 (0%)	36 (13.1%)	1 (0.7%)
	Incus absent	130 (65.7%)	185 (67.3%)	99 (64.7%)
	Stapes absent	57 (28.8%)	49 (17.8%)	42 (27.5%)
	Footplate fixation	2 (1.0%)	2 (0.7%)	2 (1.3)
	Missing	9 (4.5%)	3 (1.1%)	9 (5.9%)
Ossicular chain reconstruction	Intact chain	0 (0%)	36 (13.1%)	1 (0.7%)
	No reconstruction, chain not intact	33 (16.7%)	21 (7.6%)	14 (9.2%)
	Incus interposition	11 (5.6%)	15 (5.5%)	1 (0.7%)
	PORP	61 (30.8%)	50 (18.2%)	3 (2.0)
	TM to stapes (type III)	51 (25.8%)	115 (41.8%)	90 (58.8%)
	TORP	35 (17.7%)	12 (4.4%)	8 (5.2%)
	TM directly to footplate	1 (0.5%)	24 (8.7%)	28 (18.3%)
	Missing	6 (3.0%)	2 (0.7%)	8 (5.2%)
STAM	1	40 (20.2%)	101 (36.7%)	24 (15.7%)
	2	69 (34.8%)	79 (28.7%)	43 (31.4%)
	3	89 (44.9%)	95 (34.5%)	81 (52.9%)
Pre-operative ABG	< 20 dB HL	84 (42.4%)	141 (51.3%)	55 (35.9%)
	20–40 dB HL	92 (46.5%)	112 (40.7%)	81 (52.9%)
	> 40 dB HL	22 (11.1%)	22 (8.0%)	17 (11.1%)

*BOT* bony obliteration tympanoplasty, *CWU* canal wall up, *PORP* partial ossicular replacement prosthesis, *TM* tympanic membrane, *TORP* total ossicular replacement prosthesis, *ABG* air–bone gap

### Audiological results

The short- and long-term hearing outcomes per surgical technique in the total group of patients are given in Tables 2, 3. There were no clinically relevant differences in post-operative AC threshold, BC threshold and ABG between the short- and long-term audiometry. Due to a larger number of cases who underwent short-term audiometry, further analyses were performed using the short-term audiometry. For pre- and post-operative hearing, there was a significant difference in AC threshold, BC threshold level and ABG between the surgical techniques (*P* = 0.000, *P* = 0.000 and *P* = 0.006, respectively, for pre-operative hearing and *P* = 0.000, *P* = 0.000 and *P* = 0.000, respectively, for post-operative hearing). There were no differences in change of AC threshold, BC threshold and ABG between the different groups. There were no cases with iatrogenic sensorineural deafness.

**Table 2:** Short-term hearing outcome per surgical technique (median 14 weeks post-surgery)

*N* = 626	Air conduction						Bone conduction						Air–bone gap					
	Pre-operative median, dB HL (IQR)	*P*	Post-operative median, dB HL(IQR)	*P*	Change, dB (IQR)	*P*	Pre-operative median, dB HL(IQR)	*P*	Post-operative median, dB HL (IQR)	*P*	Change, dB (IQR)	*P*	Pre-operative median, dB (IQR)	*P*	Post-operative median, dB (IQR)	*P*	Change, dB (IQR)	*P*
BOT	42.5 (32.1–56.6)	0.000	41.8 (30.0–56.3)	0.000	– 1.3 ( – 8.8 to 6.3)	0.178	17.5 (10.9–30.0)	0.000	17.5 (11.25–27.8)	0.000	1.3 ( – 3.8 to 5.0)	0.970	22.5 (15.0–32.5)	0.006	22.5 (15.9–31.6)	0.000	0.0 ( – 6.3 to 6.6)	0.357
CWU without obliteration	37.5 (27.5–51.3)		35.0 (26.3–48.8)		1.3 ( – 3.8 to 5.0)		16.3 (10.0–25.0)		15.0 (11.3–23.8)		0.0 ( – 3.8 to 5.0)		20.0 (12.5–28.8)		18.8 (12.5–26.3)		0.0 ( – 6.3 to 7.5)	
CWD without obliteration	48.8 (34.4–62.5)		46.3 (33.8–63.1)		0.0 ( – 8.8 to 6.9)		22.5 (13.1–34.4)		21.3 (13.8–34.4)		0.0 ( – 3.4 to 5.0)		25.0 (15.0–32.5)		23.8 (16.3–33.8)		– 1.3 ( – 7.5 to 6.3)	

*dB HL* decibel hearing level, *BOT* bony obliteration tympanoplasty, *CWU*, canal wall up, *preop* pre-operative, *postop* post-operative, *IQR* inter-quartile range, *AC* air conduction, *BC* bone conduction, *ABG* air–bone gap

**Table 3:** Long-term hearing outcome per surgical technique (median 20 months post-surgery)

*N* = 560	Air conduction						Bone conduction						Air–bone gap					
	Pre-operative median, dB HL (IQR)	*P*	Post-operative median, dB HL(IQR)	*P*	Change, dB (IQR)	*P*	Pre-operative median, dB HL(IQR)	*P*	Post-operative median, dB HL (IQR)	*P*	Change, dB (IQR)	*P*	Pre-operative median, dB (IQR)	*P*	Post-operative median, dB (IQR)	*P*	Change, dB (IQR)	*P*
BOT	40.6 (30.0–55.0)	0.120	38.5 (26.3–55.6)	0.000	0.0 ( – 7.5 to 7.5)	0.065	17.5 (10.0–28.8)	0.000	18.5 (12.5–30.0)	0.000	0.0 ( – 5.0 to 3.8)	0.064	21.3 (13.8 – 28.8)	0.006	18.8 (12.5 – 27.5)	0.000	1.3 ( – 7.5 to 8.1)	0.156
CWU without obliteration	37.5 (27.5 – 51.3)		37.5 (26.3 – 48.8)		0.0 ( – 6.3 to 8.8)		17.5 (10.0 – 25.0)		16.3 (10.0 – 28.8)		0.0 ( – 6.3 to 4.1)		19.4 (12.5 – 27.5)		17.5 (10.0 – 27.5)		1.3 ( – 5.0 to 7.8)	
CWD without obliteration	48.8 (34.4 – 62.5)		48.8 (35.0 – 68.8)		– 3.8 ( – 13.8 to 7.5)		22.5 (13.1 – 34.4)		23.8 (15.0 – 38.8)		0.0 ( – 6.3 to 6.3)		25.0 (15.0 – 32.5)		25.0 (16.3 – 35.0)		– 2.5 ( – 10.0 to 7.5)	

*dB HL* decibel hearing leve, *BOT* bony obliteration tympanoplasty, *CWU* canal wall up, *preop* pre-operative, *postop* post-operative, *IQR* inter-quartile range, *AC* air conduction, *BC* bone conduction, *ABG* air–bone gap

### Subgroup analyses

Supplemental Table 1 shows univariate analyses on the impact of different variables on the change in ABG between pre- and post-operatively in the total group of patients. A positive effect size implicates relatively more patients with an improvement of ABG, whereas a negative effect size implicates relatively more patients with deterioration of ABG. The pre-operative ABG seems to have a relatively large effect size on the change in ABG and was included in the subgroup analyses for differences in post-operative ABG between the BOT and non-obliteration techniques (Table 4). Because of clinical relevance, we also included the ossicular chain reconstruction in these analyses; cases with an intact ossicular chain after cholesteatoma removal were excluded for this subgroup analyses. For all variables, no significant differences in post-operative ABG between the BOT and non-obliteration techniques were found. There were some small non-significant differences of only a few decibels per group, but they are considered clinically irrelevant.

**Table 4:** Subgroup analysis for differences in post-operative ABG between the BOT and non-obliteration techniques

		BOT		CWU and CWD without obliteration		*P*
		*N* (%)	Median post-operative ABG in dB HL (IQR)	*N* (%)	Median post-operative ABG in dB HL (IQR)	
Pre-operative ABG < 20 dB	No reconstruction, chain not intact	5	22.5 (16.3–26.9)	7	20.0 (15.0–36.3)	0.870
	Incus interposition	7	13.8 (8.8–15.0)	13	12.5 (7.5–16.9)	0.781
	PORP	30	16.3 (9.7–21.6)	25	15.0 (12.5–21.3)	0.722
	TM to stapes (type III)	22	18.8 (13.4–26.3)	91	17.5 (11.3–22.5)	0.093
	TORP	7	26.3 (15.0–33.8)	2	19.4 (16.3–NA)	0.769
	TM directly to footplate	0	NA	11	17.5 (12.5–27.5)	NA
	Total	71	17.5 (12.5–22.5)	149	16.3 (11.3–22.5)	0.471
Pre-operative ABG 20–40 dB HL	No reconstruction, chain not intact	23	31.3 (23.8–35.0)	22	28.1 (20.0–33.4)	0.351
	Incus interposition	3	18.8 (3.8–NA)	3	8.8 (6.3–NA)	0.827
	PORP	27	21.3 (16.3–31.3)	22	18.1 (13.4–26.6)	0.344
	TM to stapes (type III)	22	22.5 (19.4–34.7)	102	25.0 (17.5–32.5)	0.786
	TORP	23	23.8 (17.5–32.5)	11	20.0 (11.3–23.8)	0.090
	TM directly to footplate	1	30.0	33	28.8 (23.8–36.9)	0.878
	Total	99	23.8 (17.5–33.1)	193	25.0 (17.5–32.5)	0.975
Pre-operative ABG ≥ 40 dB HL	No reconstruction, chain not intact	5	35.0 (34.4–41.3)	6	37.5 (29.7–42.8)	1.000
	Incus interposition	1	31.3	0	NA	NA
	PORP	4	32.5 (15.0–35.0)	6	30.0 (29.5–35.0)	0.819
	TM to stapes (type III)	7	25.0 (20.0–37.5)	12	33.1 (21.3–35.9)	1.000
	TORP	5	20.0 (17.8–24.5)	7	21.3 (18.8–25.0)	0.515
	TM directly to footplate	0	NA	8	36.9 (23.8–42.2)	NA
	Total	22	30.6 (20.0–35.0)	39	30.0 (23.8–38.8)	0.498
Missing		6		10		

*BOT* bony obliteration tympanoplasty, *CWU* canal wall up, *ABG* air–bone gap, *PORP* partial ossicular replacement prosthesis, *TM* tympanic membrane, *TORP* total ossicular replacement prosthesis

Cases with intact ossicular chain were excluded

## Discussion

In this study, we evaluated and compared the hearing outcome after the BOT versus the non-obliteration CWU and CWD in a large patient cohort in our tertiary referral center. As the aeration of the middle ear is associated with hearing outcome [6, 7], we hypothesized that the post-operative hearing after the BOT may be better compared to the non-obliteration CWU and CWD technique. However, we found no significant differences in hearing outcome between those surgical techniques, but comparison of the different groups is challenging as post-operative hearing is influenced by many factors.

Since the introduction of mastoid obliteration in canal wall down (CWD) cholesteatoma surgery with canal wall reconstruction by Mercke in 1987 [17], many have reported on their outcomes after mastoid obliteration using different surgical approaches (CWU and CWD) and obliteration materials [9]. With a mean recurrent and residual cholesteatoma rate of 4.6% and 5.4%, respectively [9], the surgical outcome of obliterative techniques are overall better compared to the non-obliteration CWU and CWD in which recurrence rates (recurrent and residual cholesteatoma rate combined) of 4–70% are reported [18]. While the recurrent and residual rates after obliteration and non-obliteration cholesteatoma surgery are well described in literature, the audiological results remain underexposed as there are no comparative studies on hearing results after BOT verus CWU and CWD without obliteration. Several studies reported on hearing results after BOT or CWU/CWD without obliteration alone, in which the audiological success rate ranges from 19 to 75% (Table 5). The most common used success criterion in these studies is the percentage of cases with a post-operative ABG ≤ 20 dB, which was achieved in 19%–55.9% in the mastoid obliteration studies and 51.8–73% in the CWU/CWD without obliteration studies. In our cohort, a post-operative ABG ≤ 20 dB was achieved in 44.4% after the BOT and in 49.5% after CWU/CWD without obliteration. However, it is difficult if not impossible to systematically compare the hearing outcome of these studies as the post-operative hearing is dependent on many factors which are not commonly reported on, such as the ossicular chain status, ossicular chain reconstruction, pre-operative hearing, extension of cholesteatoma and middle ear aeration. A difference in prevalence of these factors may have caused the broad spread of results between these studies.

**Table 5:** Comparison of hearing outcome with literature

Study	Year	Population	No. of cases	Surgical technique	Success criterion	% Success	% Success in the present study with this criterion for BOT	% Success in the present study with this criterion for non-obliteration CWU/CWD
Lee et al. [19]	2005	Pediatric and adult	56	Staged CWU with obliteration	Post-operative ABG ≤ 10 dB Post-operative ABG ≤ 20 dB Post-operative ABG > 20 dB	37.4% 34.1% 28.5%	10.6% 44.4% 55.6%	16.8% 49.5% 50.5%
Gantz et al. [22]	2005	Pediatric and adult	130	CWD with obliteration	Post-operative ABG ≤ 20 dB Post-operative ABG ≤ 30 dB	19% 24%	44.4% 73.7%	49.5% 77.6%
Ajalloueyan et al. [23]	2006	All patients ≥ 14y	148	CWU and CWD	Post-operative AC ≤ 40 dB HL	42%	47.5%	55.6%
Stankovic [3]	2008	Pediatric and adult	611	CWU and CWD	Post-operative ABG ≤ 20 dB Post-operative AC ≤ 30 dB HL	51.8–68.2% 57.7–69.3%	44.4% 25.8%	49.5% 31.1%
Kang et al. [24]	2009	Adult	200	CWU with obliteration	Mean post-operative AC threshold level Mean post-operative ABG	25.3 dB HL (sd 12.2) 6.2 dB (sd 12.6)	44.8 dB HL 23.4 dB	43.2 dB HL 21.9 dB
Declerck et al. [25]	2010	Adult	161	CWU and CWD	Post-operative ABG ≤ 20 dB	57%	44.4%	49.5%
Wilson et al. [26]	2013	Pediatric and adult	156	Staged CWU	Post-operative ABG ≤ 20 dB	64%	44.4%	49.5%
Fukuda et al. [27]	2019	Adult	34	CWU and CWD with partial obliteration	Post-operative ABG ≤ 10 dB Post-operative ABG ≤ 20 dB	23.5% 55.9%	10.6% 44.4%	16.8% 49.5%
Van Waegeningh et al. [28]	2021	Adult	61	CWU with obliteration	Post-operative ABG ≤ 20 dB AC gain	51% 75%	44.4% 46.9%	49.5% 62.6%

*CWU* canal wall up, *CWD* canal wall down, *BOT* bony obliteration tympanoplasty, *ABG* air–bone gap, *AC* air conduction, *sd* standard deviation

When we compared the hearing outcome per surgical technique in the total group of patients (Tables 2, 3), we found a significant difference in AC threshold, BC threshold level and ABG while there were no differences in change of AC threshold, BC threshold and ABG between the different groups. Importantly, no conclusions can be drawn when analyzing the total group of patients due to the significant heterogenicity in patient characteristics between the treatment groups at baseline. As seen in Table 1, factors with a positive effect on improvement of hearing were more frequently seen in the CWU without obliteration group (smaller pre-operative ABG, smaller cholesteatoma according to the STAM classification). We found that the pre-operative ABG has a relatively large effect size on change in ABG between pre- and post-operative (supplemental Table 1). When stratifying for this factor along with the ossicular chain reconstruction due to clinical relevance to account for difference at baseline (Table 4), no significant differences in post-operative ABG were found between BOT and non-obliteration CWU and CWD. This was against our expectations, as we hypothesized that the post-operative hearing results after mastoid obliteration may be better compared to non-obliteration techniques. One of the rationales behind mastoid obliteration is that reducing the mucosal surface for gas exchange can result in slower gas absorption and slower pressure changes, resulting in less pathologic abnormalities of the middle ear [10]. While it is generally assumed that this results in an improved tympanic cavity aeration and thus in better hearing, the scientific evidence on this is weak and our data does not support this theory. It is stated that mastoid obliteration facilitates aeration of the middle ear [11, 19], but these studies do not compare the aeration of obliteration surgery with the aeration of non-obliteration surgery. The only comparative study on this subject was performed by Vartiainen et al. in 1987 [20], in which an adhesive tympanic membrane was seen in 12% after both CWU with obliteration and CWU without obliteration. However, in this study the epitympanic space was not obliterated, which may have caused an underestimation of the potentially positive effect of obliteration on middle ear aeration as it is suggested that the epitympanum plays an important role in the gas exchange due to highly vascularized mucosa compared to the pro-, meso- and hypotympanum [21]. Due to the retrospective design of the present study, no data on pre- and post-operative middle ear aeration were available. Further research is required to prospectively assess the role of mastoid and epitympanic obliteration on middle ear aeration.

Other explanations for the fact that we observed no differences in hearing outcome between the BOT and non-obliteration techniques may be sought in the limitations of this study. Limitations include the retrospective design, the possible bias and confounding factors and the lack of power due to relatively low patient numbers after stratification. The motives to perform a CWU or CWD without obliteration over a BOT were not always clear due to the retrospective design of the study. It is plausible that, especially in the first period after the introduction of the BOT in our institution, there has been positive selection bias for large cholesteatoma due to an adaption period for the surgeons in changing their surgical approach. Even today the CWU without obliteration is still sometimes performed at our institution in some cases of limited, primary or small cholesteatoma, while the BOT is performed in more extensive cases in which before a CWD approach would have been performed, causing a possible indication bias. As shown in Table 1 for example, the CWU without obliteration group consisted of more small cholesteatoma (STAM 1), less large cholesteatoma (STAM 3) and less cases with an absent stapes compared to the BOT group; all cases which were associated with a smaller post-operative ABG (Supplemental Table 1). We tried to minimalize the effect of this bias on our outcome by performing subgroup analyses in which we stratified for the variables with a relatively large effect size on post-operative hearing. While we believe that this was the best possible statistical method on this retrospective data, it is conceivable that the cases in the BOT group consisted of more severe or pathologic cholesteatoma compared to the non-obliteration group and that we were not able to facilitate a fair comparison between the surgical techniques. This may have resulted in worse hearing outcome in the BOT group. Furthermore, there are still several possible confounding factors which we were not able to correct for such as the infection rate, antibiotics administration, tobacco use and experience of the surgeon. The infection rate is difficult to measure, but can potentially affect the stabilization of the middle ear and thus affect hearing. Also, the experience and skill of the surgeon may affect the hearing results, but data are lacking as skill is a subjective concept. Upcoming research on audiological results after cholesteatoma surgery should therefore focus on identifying as much as possible factors which influence post-operative hearing. Finally, as we found no differences in post-operative hearing between the BOT and CWU and CWD without obliteration, we prefer the BOT to non-obliteration surgery as the recurrent and residual rates are significantly lower and the hygienic results are excellent after the former technique [9].

## Conclusion

In this large retrospective cohort study, we found no significant differences in post-operative ABG between the BOT and the non-obliteration CWU and CWD. A solid comparison of hearing between groups remains very challenging as hearing outcome seems to be dependent on many different factors. Hearing outcome seems to be no additional argument to choose for BOT over non-obliteration surgery.

## Supplementary Information

Below is the link to the electronic supplementary material.

Supplementary file1 (DOCX 14 KB)
