# Supplementary material for: A retrospective analysis of hearing after cholesteatoma surgery: the bony obliteration tympanoplasty versus canal wall up and canal wall down without mastoid obliteration
Source: Eur Arch Otorhinolaryngol. 2022 Apr 10;279(11):5181–9. doi: 10.1007/s00405-022-07367-x (PMC9519705; doi:10.1007/s00405-022-07367-x)
Supplement: Supplementary file 1 — Supplementary file1 (DOCX 14 KB) [file 405_2022_7367_MOESM1_ESM.docx]

**SUPPLEMENTAL DATA**

Supplemental table 1. Effect size on change in ABG

|  | | Effect size on change in ABG |
| --- | --- | --- |
| **Sex** | Female | 0,16 |
|  | Male | 0,06 |
| **Side** | Left | 0,10 |
|  | Right | 0,09 |
| **Primary / revision** | Primary | -0,02 |
|  | Revision | 0,20 |
| **Ossicular chain before cholesteatoma removal** | Intact chain | -0,20 |
|  | Incus absent | 0,13 |
|  | Stapes absent | 0,21 |
|  | Footplate fixation | -0,67 |
|  | Missing |  |
| **Ossicular chain after cholesteatoma removal** | Intact chain | -0,12 |
|  | Incus absent | 0,08 |
|  | Stapes absent | 0,15 |
|  | Footplate fixation | -0,67 |
|  | Missing |  |
| **Ossicular chain reconstruction** | Intact chain | -0,12 |
|  | No reconstruction, chain not intact | 0,08 |
|  | Incus interposition | 0,22 |
|  | PORP | 0,14 |
|  | TM to stapes (type III) | 0,05 |
|  | TORP | 0,33 |
|  | TM directly to footplate | -0,23 |
|  | Missing |  |
| **STAM** | 1 | 0,18 |
|  | 2 | 0,15 |
|  | 3 | 0,00 |
| **Pre-operative ABG** | <20 dB HL | -0,27 |
|  | 20 – 40 dB HL | 0,29 |
|  | >40 dB HL | 0,72 |

PORP, partial ossicular replacement prosthesis; TM, tympanic membrane; TORP, total ossicular replacement prosthesis; ABG, air-bone gap

A negative effect size indicates relatively more patients with a deterioration of ABG, a positive effect size implicates relatively more patients with an improvement of ABG.

An effect size between 0.10 and 0.30 was indicated as a small effect size, 0.30 - 0.50 as a medium effect size, and > 0.50 as a large effect size^16^
